# 20‐year depressive symptoms, dementia, and structural neuropathology in older women

**DOI:** 10.1002/alz.13781

**Published:** 2024-04-09

**Authors:** Andrew J. Petkus, Xinhui Wang, Diana Younan, Lauren E. Salminen, Susan M. Resnick, Stephen R. Rapp, Mark A. Espeland, Margaret Gatz, Keith F. Widaman, Ramon Casanova, Helena Chui, Ryan T. Barnard, Sarah A. Gaussoin, Joseph S. Goveas, Kathleen M. Hayden, Victor W. Henderson, Bonnie C. Sachs, Santiago Saldana, Aladdin H. Shadyab, Sally A. Shumaker, Jiu‐Chiuan Chen

**Affiliations:** ^1^ Department of Neurology University of Southern California Los Angeles California USA; ^2^ Department of Population and Public Health Sciences University of Southern California Los Angeles California USA; ^3^ Mark and Mary Stevens Neuroimaging and Informatics Institute University of Southern California Marina del Rey California USA; ^4^ Laboratory of Behavioral Neuroscience National Institute on Aging Baltimore Maryland USA; ^5^ Department of Neurology Wake Forest School of Medicine Winston‐Salem North Carolina USA; ^6^ Department of Social Sciences and Health Policy Wake Forest School of Medicine Winston‐Salem North Carolina USA; ^7^ Department of Biostatistics and Data Sciences Wake Forest School of Medicine Winston‐Salem North Carolina USA; ^8^ Department of Internal Medicine Wake Forest School of Medicine Winston‐Salem North Carolina USA; ^9^ Center for Economic and Social Research University of Southern California Los Angeles California USA; ^10^ Graduate School of Education University of California, Riverside Riverside California USA; ^11^ Department of Psychiatry and Behavioral Medicine Medical College of Wisconsin Milwaukee Wisconsin USA; ^12^ Departments of Epidemiology and Population Health and of Neurology and Neurological Sciences Stanford University Palo Alto California USA; ^13^ Herbert Wertheim School of Public Health and Human Longevity Science University of California, San Diego La Jolla California USA

**Keywords:** Alzheimer's disease neurodegeneration, cerebrovascular disease, dementia, depression, joint modeling, women

## Abstract

**INTRODUCTION:**

The course of depressive symptoms and dementia risk is unclear, as are potential structural neuropathological common causes.

**METHODS:**

Utilizing joint latent class mixture models, we identified longitudinal trajectories of annually assessed depressive symptoms and dementia risk over 21 years in 957 older women (baseline age 72.7 years old) from the Women's Health Initiative Memory Study. In a subsample of 569 women who underwent structural magnetic resonance imaging, we examined whether estimates of cerebrovascular disease and Alzheimer's disease (AD)‐related neurodegeneration were associated with identified trajectories.

**RESULTS:**

Five trajectories of depressive symptoms and dementia risk were identified. Compared to women with minimal symptoms, women who reported mild and stable and emerging depressive symptoms were at the highest risk of developing dementia and had more cerebrovascular disease and AD‐related neurodegeneration.

**DISCUSSION:**

There are heterogeneous profiles of depressive symptoms and dementia risk. Common neuropathological factors may contribute to both depression and dementia.

**Highlights**

- The progression of depressive symptoms and concurrent dementia risk is heterogeneous.
- Emerging depressive symptoms may be a prodromal symptom of dementia.
- Cerebrovascular disease and AD are potentially shared neuropathological factors.

## 1 BACKGROUND

Late‐life depressive symptoms and all‐cause dementia are public health concerns that are frequently comorbid and disproportionately impact women.^1^, ^2^ The interrelationship between depressive symptoms and clinical dementia onset in later life is inconclusive, and complex, with heterogeneous patterns.^3^, ^4^ Depressive episodes may be a causal factor exerting a neurotoxic effect on the brain, thereby increasing dementia risk.^5^ Meta‐analyses report that individuals with a past major depressive episode are 1.85 times more likely to develop all‐cause dementia, 1.65 times more likely to develop Alzheimer's dementia, and 2.52 times more likely to develop vascular dementia.^6^ Alternatively, and not mutually exclusive, depressive symptoms might be a prodromal manifestation of the underlying neurodegenerative process leading to dementia as multiple pathophysiological factors, including inflammation, cerebrovascular disease,^7^, ^8^ and Alzheimer's disease (AD)‐related neurodegeneration, are common to the etiology of both late‐life depressive symptoms and dementia.^5^, ^9^ Understanding the heterogeneous patterns of depressive symptoms and dementia risk across older adulthood, and the underlying pathophysiological processes driving this association will provide important insight into clinical care to prevent incident future dementia and the development of depressive symptoms in older adulthood.

The initial age of onset of depressive symptoms may explain some of the likely heterogeneity in the interrelationship between late‐life depressive symptoms and dementia risk.^10^, ^11^ Early‐onset late‐life depression, defined here as a depressive episode in early or mid‐life, is likely due to genetic factors, early‐life experiences, sex‐hormonal factors, and substance use.^12^ Individuals with early‐onset depression are more likely to experience a recurrence of depressive symptoms in older adulthood.^13^ The recurrent nature of early‐life onset depressive symptoms may exert cumulative neurotoxic effects contributing to accelerated brain aging, increased allostatic load, hippocampal atrophy via prolonged glucocorticoid activation, and accumulation of cerebrovascular disease.^13^ Thus, late‐life depressive symptoms with onset before older adulthood likely represent a causal factor of dementia in later life. Alternatively, late‐life depressive symptoms with initial onset in older adulthood likely represent a prodromal symptom of incident vascular or neurodegenerative disease.^14^

Data‐driven approaches to explain the heterogeneity between depressive symptom trajectories and dementia risk can inform future research and improve clinical care by identifying unique phenotypes of individuals who have similar longitudinal progression of depressive symptoms and dementia risk across later life. Studies utilizing data‐driven latent trajectory approaches to classify the longitudinal progression of depressive symptoms report that individuals endorsing increases in depressive symptoms over 5‐^15^ and 11‐year^16^ periods were at higher risk for developing dementia over a subsequent follow‐up period. These studies have methodological limitations including (1) failing to account for changes in depressive symptoms up until dementia onset and (2) assessing depressive symptoms over time frames (5 and 11 years) that are short relative to the lengthy preclinical phase of dementia. A joint modeling approach,^17^ where depressive symptom trajectories and dementia risk are modeled concurrently, may address the limitations of previous research by categorizing the heterogeneity both in the progression of depressive symptoms over time and the risk of dementia. Furthermore, it is unknown whether neuropathological factors (eg, AD‐related neurodegeneration or cerebrovascular disease) and age of onset are differentially associated with the heterogeneous courses of depressive and dementia risks.

Here, we conducted a longitudinal study spanning 21 years, where we applied a joint modeling approach to identify groups of women who have similar trajectories of annually assessed depressive symptoms across mid‐to‐late‐older adulthood and concurrent risk of dementia in late‐older adulthood. Our primary aim was to identify heterogeneous trajectories of depressive symptoms and dementia risk. Our secondary aim was to examine whether structural magnetic resonance imaging (MRI) estimates of global white matter small‐vessel ischemic disease (WM‐SVID) or AD‐related neurodegeneration measured during the preclinical phase underlie the identified trajectories.

## 2 METHODS

### 2.1 Study population

**FIGURE 1 alz13781-fig-0001:**
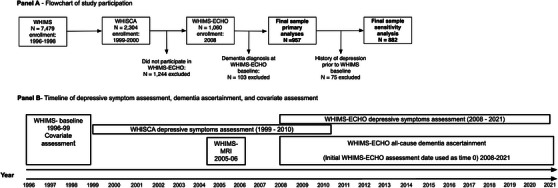
Flowchart of study participation (Panel A) and timeline of study assessments (Panel B).

This study included 957 community‐dwelling older women (aged 72.7 ± 3.5 years old at baseline) who completed annual assessments of depressive symptoms through participation in both the Women's Health Initiative Study of Cognitive Aging (WHISCA; study years 1999 to 2010)^18^ and the Women's Health Initiative Memory Study (WHIMS) of the Epidemiology of Cognitive Health Outcomes (WHIMS‐ECHO; study years 2008 to 2021).^19^ WHISCA was an ancillary study to WHIMS,^13^ which was itself an ancillary study to the larger Women's Health Initiative (WHI) trial of postmenopausal hormone therapy.^20^ A subsample of study participants (*n* = 569) underwent structural magnetic resonance imaging (sMRI) of the brain in 2005 to 2006.^21^ All women were dementia‐free at the WHIMS‐ECHO baseline. Annual WHIMS‐ECHO depression assessments available through 2018 were used in the current study. See Figure 1A for a flowchart of study participation and Figure 1B for a timeline of study participation.

**RESEARCH IN CONTEXT**

1. **Systematic review**: PubMed was used to identify studies that examined the longitudinal course of depressive symptom severity and dementia risk. No studies were found that utilized a joint modeling approach to identify heterogeneous subgroups of women who exhibited similar progression of depressive symptoms and concurrent dementia risk over a 20‐year follow‐up period.
2. **Interpretation**: Results show that women who reported emerging depressive symptoms (assessed annually) over the follow‐up period were at the highest risk of developing dementia. Women with increasing depressive symptoms also had more cerebrovascular disease and AD ‐related neurodegeneration relative to women with minimal symptoms.
3. **Future directions**: Findings highlight the utility of joint modeling approaches to elucidate heterogeneous concurrent progression of depressive symptoms and dementia risk across older adulthood. More research is needed to examine the role of cerebrovascular disease and AD as a common factor contributing to both depressive symptoms and dementia risk.

### 2.2 Assessment of depressive symptoms

Depressive symptoms were assessed at each WHISCA and WHIMS‐ECHO evaluation (range 5 to 19 assessments) using the 15‐item Geriatric Depression Scale (GDS‐15).^22^ The GDS‐15 was administered in person during WHISCA assessments and over the phone during WHIMS‐ECHO assessments. Scores range from 0 to 15 and the GDS‐15 has similar reliability and validity when administered in‐person or over the telephone.^23^ A score of 5 or higher is the suggested cutoff to represent clinically significant depressive symptoms. Participants were coded as having a history of depression if they stated yes to both of the depression questions from the National Institute of Mental Health Diagnostic Interview Schedule^24^ at the WHIMS baseline that took place between 1996 and 1999.

### 2.3 Classification of dementia

Incident cases of all‐cause dementia were defined using the Diagnostic and Statistical Manual of Mental Disorders, Fourth Edition (DSM‐IV) criteria. During WHIMS participation (1996 to 2008), women completed annual screening of cognitive performance through administration of the Modified Mini‐Mental State (3MS) examination.^25^ Women who scored below an education‐adjusted cut point on the 3MS completed an in‐person comprehensive evaluation of cognitive function, neuropsychological and functional assessment, and collection of clinical data to rule out possible reversible causes of cognitive decline.^26^ During WHIMS‐ECHO evaluations (2008 to 2021), cognitive assessments were conducted by telephone using a validated battery that included the Telephone Interview for Cognitive Status‐Modified (TICS‐m).^27^ For those women with TICS‐m below 31, a reliable informant was contacted and a structured interview (the Dementia Questionnaire)^28^ was completed. The same central adjudication process was used in WHIMS and WHIMS‐ECHO to identify dementia.

### 2.4 All‐cause mortality

All‐cause mortality, up to 2021, was examined as a competing risk in time‐to‐event analyses. The WHI data were linked with the National Death Index of the National Center for Health Statistics to identify the date on which individuals died.

### 2.5 Structural MRI assessment

Participants underwent a[Fig alz13781-fig-0001] sMRI brain scan on 1.5T scanners at one of 14 WHIMS clinical centers using a standardized protocol developed by the WHIMS‐MRI Quality Control Center at the University of Pennsylvania.^21^, ^29^ The scan series for volumetric imaging used a 22‐cm field of view and a 256 × 256 acquisition matrix and the following pulse sequences: oblique axial spin density/T2‐weighted spin‐echo images, oblique axial fast fluid‐attenuated inversion recovery (FLAIR) T2‐weighted spin echo image, and oblique axial fast spoiled 3D T1‐weighted gradient echo images. Details on the pulse sequence parameters were published previously.^21^, ^29^

#### 2.5.1 Assessment of AD‐related neurodegeneration

Machine learning‐derived AD pattern similarity (AD‐PS) scores were used to estimate the magnitude of AD‐related neurodegeneration. AD‐PS scores are conditional probability metrics (ranging from 0 to 1) that reflect the degree of spatial similarity between a participant's gray matter and the pattern of gray matter in individuals with AD. Scores were calculated using data‐driven methods developed and validated using the Alzheimer's Disease Neuroimaging Initiative (ADNI) data. Detailed technical information is provided elsewhere.^30^ Higher AD‐PS scores indicate higher AD‐related neurodegeneration.

#### 2.5.2 Assessment of global small‐vessel ischemic disease

White matter lesions (WML) were segmented using a deep learning‐based machine learning algorithm that differentiated WML segmentation from cases with highly variable WM‐SVID.^31^ White matter was classified as normal or abnormal, and abnormal voxels were summed across regions to represent a global measure of WM‐SVID volume.

### 2.6 Assessment of covariates

A structured questionnaire was administered at the WHIMS baseline visit to gather information on demographics (age, race/ethnicity), geographic region of residence (Northeast, South, Midwest, and West), socioeconomic status (education, family income, employment status), and lifestyle factors (smoking; alcohol use, physical activity). Clinical characteristics were ascertained, including postmenopausal hormone treatment, history of cardiovascular disease (including previous coronary heart, stroke, or transient ischemic attack), hypertension (defined as elevated blood pressure or use of antihypertensive medication), hypercholesteremia, and diabetes mellitus (defined as physician diagnosis plus oral medications, or insulin therapy). The self‐reported medical histories and the physical measures are reliable and valid.^32^

### 2.7 Statistical analysis

Joint latent class mixture models (JLCMMs)^17^, ^33^ were constructed to identify individuals who exhibited similar patterns of depressive symptoms across mid‐to‐late older adulthood. The JLCMM allows for simultaneous analysis of (1) a longitudinal continuous marker (eg, depressive symptoms up to and before the clinical event of interest) and (2) the time to event of interest (eg, dementia and non‐dementia‐related mortality). The first component of the JLCMM was a mixed‐effect regression with class‐specific estimates of the intercept and slope, which characterized the longitudinal trajectory of the GDS‐15 over the study period. Assessments of depressive symptoms that took place after a dementia diagnosis were excluded from these analyses. Years across repeated GDS‐15 assessments were used as the time scale, which was centered on the individual‐specific day of the WHIMS‐ECHO baseline. A piecewise‐linear model with random effect of the intercept was fit where two linear slope parameters were estimated for each latent class. Women were predominantly between 73 and 80 years old during the WHISCA study period and 80 years and older during the WHIMS‐ECHO period. We therefore refer to the WHISCA study period as mid‐older adulthood and the WHIMS‐ECHO study period as late‐older adulthood. The first linear slope parameter captured the linear change in depressive symptoms during mid‐older adulthood. The second linear slope parameter captured the linear change in depressive symptoms during late‐older adulthood. The intercept of the model represents estimated depressive symptoms at mid to late older adulthood (eg, WHIMS‐ECHO baseline) inflection point. We chose this time metric (years since WHIMS‐ECHO baseline) and centering point (day of WHIMS‐ECHO baseline) to account for the two different study protocols (WHISCA was an in‐person assessment while WHIMS‐ECHO was a telephone‐based assessment). Second, all women were dementia‐free throughout the entire WHISCA study period and at the WHIMS‐ECHO baseline. Estimating trajectories across this period when no individuals have dementia resembles the two‐step approach utilized by previous studies using latent class mixture models to examine associations between depressive symptom trajectories and dementia risk. Under the two‐step approach, trajectories of depressive symptoms were identified in one model followed by a second analysis where the relative risk of dementia was examined over a second follow‐up period (ie, not concurrently). Estimating a piecewise linear model with a slope parameter for each study allowed us to make inferences regarding the benefits of the JLCMM over the conventional two‐step approach. The piecewise linear model adjusted for basic sociodemographic covariates (eg, age at WHISCA baseline, education, region of residence, household income, and race/ethnicity). A three‐quantile spline transformation was applied to the GDS‐15 to account for the skewness inherent in depressive symptom data.[Fig alz13781-fig-0002]

The second component of the JLCMM was a Cox regression with a two‐parameter Weibull baseline hazard function for both dementia and non‐dementia mortality. The survival time was calculated as the number of years from the WHIMS‐ECHO baseline to the date of first dementia diagnosis, death, last assessment, or June 2021, whichever occurred first. The survival models adjusted for age at the WHISCA baseline, education, region of residence, household income, and race/ethnicity.

The JLCMM links the linear mixed‐effect regression and the time‐to‐event components via the identification groups of women, referred to as latent classes who exhibit similar longitudinal profiles of depressive symptoms and dementia risk across the entire study period. The latent classes are probabilistic in that each woman is assigned a posterior probability of classification into the respective identified latent classes. We first fit a one‐class model and sequentially increased the number of latent classes. The decision on the total number of latent classes to be retained was made by a combination of the overall model fit as evidenced by the smaller Bayesian Information Criterion (BIC), the posterior probabilities, and the interpretability of the latent classes. Because the goal of these analyses was to identify heterogeneous latent classes of women who have similar patterns of depressive symptoms and dementia risk, we only included essential demographic covariates in the models, as is standard in JLCMM modeling. Covariates are limited in this way because adding covariates in the linear mixed model or the survival analyses may impact latent class membership. The JLCMMs were fitted with the Latent Class Mixed Models (LCMM)^33^ package in R.

After estimating latent class membership, we compared each class by various demographic, geographic, socioeconomic, lifestyle, and clinical variables measured at the WHI baseline using ANOVA for continuous and chi‐squared tests for categorical variables. In the subsample that underwent structural neuroimaging, weighted multivariable multinomial logistic regression models were constructed to examine whether AD‐related neurodegeneration and WM‐SVID burden were associated with latent class membership. The latent class with the baseline hazard dementia risk and fewest depressive symptoms was used as the reference group in these analyses. Models adjusted for sociodemographic (age at WHISCA baseline, race/ethnicity, region of residence, employment status, household income), lifestyle (smoking, alcohol use, physical activity), and clinical covariates (cardiovascular disease, hypertension, hypercholesterolemia, diabetes, hormone use, and hormone therapy assignment). To account for the uncertainty in latent class membership, the posterior probability of latent class membership was included in the model as a weight.

Two sensitivity analyses were conducted. First, we fitted the JLCMM models with chronological age at each assessment modeled as time in the mixed model regression component of the JLCMM. The purpose of this sensitivity analysis was to test the robustness of our findings with alternative metrics of time. In this sensitivity analysis, we fitted a piecewise linear model centered at age 80. The first slope parameter estimated linear change in depressive symptoms before age 80. The second slope parameter estimated linear change after age 80. The intercept of the model represented estimated depressive symptoms at age 80. We chose the centering point of age 80 as the average age at the WHIMS‐ECHO baseline was just over 80 years old. A second sensitivity analysis was conducted to examine whether a history of depression before older adulthood was driving the observed findings. In these sensitivity analyses, we ran all analyses excluding the 75 women who had a self‐reported history of depression before the WHIMS baseline.

## 3 RESULTS

### 3.1 Participant characteristics

Most of these older women were non‐Hispanic White (91.4%) and had more than a high school education (75.1%). Throughout the study period, participants completed an average of 14.4 (SD = 2.91; range = 5 to 19) annual assessments of depressive symptoms.

### 3.2 Trajectories of depressive symptoms

**FIGURE 2 alz13781-fig-0002:**
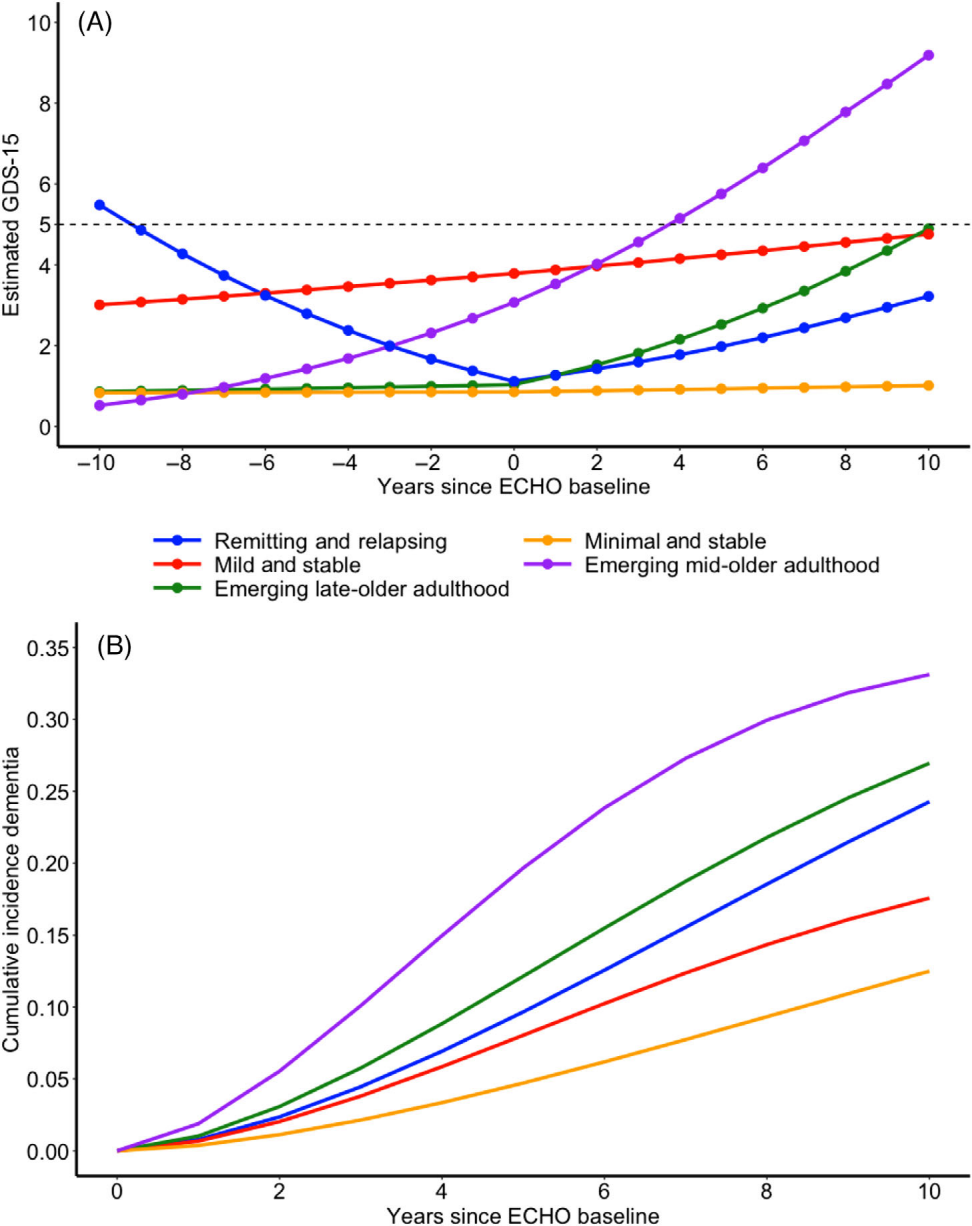
Graph of estimated mean score of 15‐item Geriatric Depression Scale over time for each joint latent class (Panel A) and cumulative incidence of dementia (Panel B) for each latent class of depressive symptoms. The dashed line in Panel A represents the recommended cutoff suggestive of clinically elevated depressive symptoms.

We identified five significant latent classes of women with similar trajectories of depressive symptoms and dementia risk using the JLCMM approach (see Table S1 for model fit statistics and Table S2 for intercept and slope estimates). Panel A in Figure 2 depicts the estimated depressive symptoms (transformed back to GDS‐15 units) for each latent class over time from the five‐class solution. The largest class consisted of women reporting minimal depressive symptoms that were stable across both study periods (minimal and stable: 36%; *n* = 343). The second largest class (mild and stable: 25%; *n* = 237) reported mild depressive symptoms that were just under the suggested cutoff for clinically significant depressive symptoms and stable across both study periods. The third class included 44 women (remitting and relapsing: 5%) with initially clinically significant depressive symptoms that decreased during mid‐older adulthood followed by an increase in later‐older adulthood. A fourth class consisted of 90 women (emerging mid‐older adulthood: 9%) with minimal depressive symptoms at baseline that emerged during both mid and late‐older adulthood. Women in this latent class had estimated depressive symptoms in the clinically significant range approximately 4 years into the WHIMS‐ECHO study period (Figure 2 Panel A). Last, a fifth class consisted of 243 women (emerging late‐older adulthood: 25%) who reported minimal symptoms across mid‐older adulthood with increasing depressive symptoms over late‐older adulthood. Despite the increase in depressive symptoms across the WHIMS‐ECHO period the overall level of symptoms remained below the suggested clinical cutoff of five. Table S3 presents the effects of covariates on the level of depressive symptoms. Non‐Hispanic White women and women with higher household incomes tended to have lower depressive symptoms compared to their counterparts.

### 3.3 Comparisons of baseline characteristics between classes

Table 1 presents a comparison of baseline characteristics by the five identified latent classes. Women reporting mild and stable depressive symptoms were more likely to have lower household incomes relative to the other latent classes. Last, women with mild and stable depressive symptoms tended to have higher rates of hypertension and diabetes and were more likely to report engaging in no moderate or strenuous physical activity compared to other classes.

**TABLE 1:** Comparison of population characteristics at Women's Health Initiative Study of Cognitive Aging (WHISCA) baseline by joint latent class^a^ of depressive symptoms and dementia risk (*N* = 957).

	Minimal and stable		Mild and stable		Remitting and relapsing		Emerging mid‐older adulthood		Emerging late‐older adulthood		
	*n* = 343		*n* = 237		*n* = 44		*n* = 90		*n* = 243		
Variable	Mean or percentage^b^	(SD) or (*N*)	Mean or percentage^b^	(SD) or (*N*)	Mean or percentage^b^	(SD) or (*N*)	Mean or percentage^b^	(SD) or (*N*)	Mean or percentage^b^	(SD) or (*N*)	*P* value^c^
Baseline GDS‐15^d^	0.50	(1.01)	2.69	(2.42)	4.55	(2.98)	0.54	(1.12)	0.60	(1.09)	**<0.001**
History of depression											**<0.001**
No	37%	(330)	22%	(193)	4%	(39)	10%	(87)	26%	(233)	
Yes	18%	(13)	58%	(43)	7%	(5)	4%	(3)	14%	(10)	
Number of assessments	14.69	(2.96)	12.80	(3.29)	14.98	(3.04)	11.19	(2.57)	13.34	(3.07)	**<0.001**
Baseline age (years)	72.03	(3.19)	73.18	(3.66)	71.75	(3.16)	73.54	(3.66)	73.06	(3.51)	**<0.001**
Region of residence, %											0.150
Northeast	31%	(66)	25%	(54)	5%	(11)	13%	(28)	26%	(55)	
South	34%	(50)	22%	(33)	3%	(4)	14%	(20)	27%	(40)	
Midwest	40%	(141)	23%	(80)	5%	(18)	7%	(24)	25%	(87)	
West	35%	(86)	28%	(70)	4%	(11)	7%	(18)	25%	(61)	
Race, %											0.125
Non‐Hispanic White	36%	(311)	25%	(214)	4%	(35)	10%	(84)	26%	(222)	
Minority	35%	(32)	25%	(23)	10%	(9)	7%	(6)	23%	(21)	
Education %											**0.011**
Less than high school	19%	(7)	42%	(15)	0%	(0)	3%	(1)	36%	(13)	
High school	36%	(73)	23%	(46)	5%	(11)	14%	(29)	22%	(44)	
More than high school	37%	(263)	25%	(176)	5%	(33)	8%	(60)	26%	(186)	
Household income											**0.006**
Less than $19,999	28%	(50)	34%	(62)	4%	(7)	12%	(21)	22%	(40)	
$20,000 to $34,999	34%	(96)	24%	(68)	5%	(15)	11%	(31)	25%	(71)	
$35,000 to $49,999	36%	(78)	24%	(51)	5%	(10)	8%	(18)	27%	(58)	
$50,000 to $74,999	51%	(78)	18%	(28)	5%	(8)	4%	(6)	22%	(33)	
$75,000 or more	32%	(32)	20%	(20)	2%	(2)	12%	(12)	34%	(34)	
Missing or don't know	32%	(9)	29%	(8)	7%	(2)	7%	(2)	25%	(7)	
Lifestyle											
Smoking, %											0.145
Never smoked	39%	(206)	22%	(116)	5%	(29)	9%	(48)	25%	(134)	
Past smoker	32%	(124)	28%	(107)	3%	(12)	10%	(39)	26%	(100)	
Current Smoker	31%	(11)	33%	(12)	8%	(3)	6%	(2)	22%	(8)	
Missing	33%	(2)	33%	(2)	0%	(0)	17%	(1)	17%	(1)	
Alcohol, %											**0.001**
Non‐drinker	37%	(41)	17%	(19)	5%	(6)	9%	(10)	31%	(34)	
Past drinker	23%	(36)	33%	(51)	6%	(9)	16%	(25)	22%	(35)	
< = 1 drink per day	37%	(211)	25%	(145)	5%	(27)	8%	(44)	25%	(143)	
>1 drink per day	46%	(54)	19%	(22)	2%	(2)	8%	(10)	25%	(30)	
Missing	33%	(1)	0%	(0)	0%	(0)	33%	(1)	33%	(1)	
Moderate or strenuous physical activity ≥20 min											**0.022**
No activity	32%	(168)	29%	(152)	6%	(29)	9%	(45)	25%	(132)	
Some activity	27%	(15)	29%	(16)	4%	(2)	9%	(5)	31%	(17)	
2 to 4 episodes/week	41%	(81)	20%	(40)	4%	(8)	10%	(20)	26%	(51)	
>4 episodes/week	45%	(79)	16%	(29)	3%	(5)	11%	(20)	24%	(43)	
Clinical											
Hypertension, % (*n*)											0.251
No	38%	(241)	23%	(145)	5%	(29)	10%	(62)	25%	(157)	
Yes	32%	(101)	28%	(90)	5%	(15)	9%	(28)	27%	(85)	
Missing	25%	(1)	50%	(2)	0%	(0)	0%	(0)	25%	(1)	
Cardiovascular disease, % (*n*)											**0.011**
No	38%	(310)	23%	(191)	5%	(39)	9%	(77)	25%	(208)	
Yes	23%	(28)	35%	(43)	5%	(5)	10%	(12)	28%	(34)	
Missing	50%	(5)	30%	(3)	0%	(0)	10%	(1)	10%	(1)	
Diabetes, % (*n*)											**0.002**
No	37%	(341)	24%	(221)	5%	(42)	10%	(88)	25%	(234)	
Yes	6%	(2)	52%	(16)	6%	(2)	6%	(2)	29%	(9)	
Hypercholestermia, % (*n*)											0.490
No	36%	(188)	24%	(188)	5%	(36)	10%	(80)	25%	(196)	
Yes	33%	(47)	30%	(47)	4%	(7)	5%	(8)	28%	(44)	
Missing	38%	(2)	15%	(2)	8%	(1)	15%	(2)	23%	(3)	
Hormone use ever, % (*n*)											**0.004**
No	37%	(192)	20%	(103)	5%	(25)	9%	(46)	29%	(147)	
Yes	34%	(151)	30%	(134)	4%	(19)	10%	(44)	22%	(96)	
Hormone treatment assignment, % (*n*)											**0.032**
Estrogen‐alone intervention	34%	(62)	34%	(63)	3%	(5)	9%	(17)	21%	(38)	
Estrogen‐alone control	31%	(55)	29%	(51)	4%	(7)	11%	(20)	24%	(43)	
Estrogen+Progestin intervention	40%	(115)	20%	(57)	4%	(12)	9%	(26)	26%	(74)	
Estrogen+Progestin control	36%	(111)	21%	(66)	6%	(20)	9%	(27)	28%	(88)	

*Note*: Bolded estimated denote *p* < 0.05.

^a^ Group membership derived from joint latent class model examining trajectories of depressive symptoms and competing risks of dementia and nondementia mortality.

^b^ Percentages reported represent row percentages.

^c^ *P* values calculated from chi‐squared or Fisher exact tests for categorical variables, from ANOVA *F*‐tests for continuous variables.

^d^ GDS‐15 = Geriatric Depression Scale‐15.

### 3.4 Risk of dementia for separate trajectories

The class‐specific cumulative incidence of all‐cause dementia from the covariate‐adjusted five‐class JLCMM is presented in Panel B of Figure 2. Using women with minimal depressive symptoms as the reference, women with depressive symptoms that began to emerge in mid‐older adulthood had the highest risk of developing dementia (Hazard Ratio (HR) = 5.10; 95% confidence interval [CI] = 2.63, 9.88; *p* < 0.001; Table 2) in late‐older adulthood. Women with increasing depressive symptoms during late‐older adulthood were also significantly more likely to develop dementia concurrently during late‐older adulthood (HR = 2.78; 95% CI = 1.48, 5.19; *p* = 0.001). Women reporting mildly elevated depressive symptoms, just below the clinical cutoff of 5, that were stable throughout both study periods were marginally more likely to develop dementia during late older adulthood (HR = 1.82; 95% CI = 0.99, 3.37; *p* = 0.054). Women reporting relapsing and remitting depressive symptoms were not at significantly higher risk of dementia compared to women with minimal depressive symptoms throughout the study period. Table S3 presents the effects of covariates on dementia risk. Women who were older at the WHISCA baseline and had lower incomes were more likely to develop dementia compared to their counterparts.

**TABLE 2:** Risk of dementia over the Women's Health Initiative Memory Study Epidemiology of Cognitive Health Outcomes (WHIMS‐ECHO) study period by identified joint latent class relative to women with minimal symptoms throughout the study period (*N* = 957).

	Outcome: Incident dementia			
Joint latent class^a^	Percentage (*n*)	HR^b^	95% CI	*P* value
Minimal and stable (*n* = 343)	11% (38)	1.00	Ref	Ref
Mild and stable (*n* = 237)	19% (45)	1.82	[0.99, 3.37]	0.054
Relapsing and remitting (*n* = 44)	23% (10)	2.11	[0.88, 5.03]	0.100
Emerging mid‐older adulthood (*n* = 90)	**30% (27)**	**5.10**	**[2.63, 9.88]**	**<0.001**
Emerging late‐older adulthood (*n* = 243)	**30% (73)**	**2.78**	**[1.48, 5.19]**	**0.001**

*Note*: Bolded estimates denote *p* < .05.

Abbreviations: CI, confidence interval; HR, Hazard Ratio; Ref, reference group.

^a^ Group membership derived from joint latent class model examining trajectories of depressive symptoms and competing risks of dementia and nondementia mortality.

^b^ The effect estimates in the partially adjusted model are adjusted for age at Women's Health Initiative Study of Cognitive Aging baseline, race/ethnicity, region of residence, household income, and education.

### 3.5 Structural MRI analysis

Among the subsample of 569 women with sMRI data, multinomial logistic regression analyses revealed that women with higher WM‐SVID (per SD) were more likely to report depressive symptoms that began to emerge in mid‐older adulthood (odds ratio [OR] = 1.58; 95% CI = 1.11, 2.26; *p* = 0.012, Table 3) relative to women with minimal depressive symptoms throughout the study period. Higher WM‐SVID was also associated with significantly increased odds of being classified as having mild and stable symptoms across the study period (OR = 1.36; 95% CI = 1.00, 1.85; *p* = 0.047). With the minimal stable depressive symptom class as the referent, higher AD‐PS (per SD) was associated with increased odds of endorsing mild and stable symptoms throughout the study period (OR = 1.48; 95% CI = 1.05, 2.11; *p* = 0.027) or depressive symptoms that emerged in late‐older adulthood (OR = 1.82; 95% CI = 1.31, 2.52; *p* < 0.001), but not with other latent classes. Additionally, self‐reporting a history of depression before the WHIMS baseline was associated with increased odds of endorsing mild and stable symptoms throughout both study periods (OR = 7.89; 95% CI = 2.59, 24.03; *p* < 0.001).

**TABLE 3:** Weighted^a^ multivariable multinomial logistic regressions to examine the effect of white matter small‐vessel ischemic disease and Alzheimer's disease‐like neurodegeneration on probability of being classified into respective joint latent class^b^ (*N* = 569).

Outcome: Joint latent class membership	WM‐SVID	WM‐SVID effect per SD			AD‐PS	AD‐PS effect per SD		
	Mean (SD)	OR^c^	95% CI	*p*	Mean (SD)	OR^c^	95% CI	*P* value
Minimal and stable (*n* = 213)	2.56 (3.64)	1.00	Ref	Ref	0.23 (0.16)	1.00	Ref	Ref
Mild and stable (*n* = 133)	4.17 (5.19)	**1.36**	**[1.00, 1.85]**	**0.047**	0.31 (0.19)	**1.48**	**[1.05, 2.11]**	**0.027**
Relapsing and remitting (*n* = 29)	2.13 (2.92)	0.79	[0.39, 1.62]	0.526	0.27 (0.17)	1.61	[0.92, 2.82]	0.095
Emerging mid‐older adulthood (*n* = 47)	5.17 (6.90)	1.58	**[1.11, 2.26]**	**0.012**	0.31 (0.17)	1.42	[0.89, 2.28]	0.145
Emerging late‐older adulthood (*n* = 147)	2.87 (3.33)	1.07	[0.77, 1.50]	0.669	0.33 (0.22)	**1.82**	**[1.31, 2.52]**	**<0.001**

*Note*: Bolded terms denote *p* < 0.05.

Abbreviations: AD‐PS, Alzheimer's disease pattern similarity score; CI, confidence interval; OR, odds ratio; Ref, reference group; WM‐SVID, white matter small‐vessel ischemic disease.

^a^ To account for the uncertainty in latent class membership, the posterior probability of latent class membership was included as a weight in the multivariable multinomial logistic regression.

^b^ Group membership derived from the joint latent class model examining trajectories of depressive symptoms and competing risks of dementia and non‐dementia mortality.

^c^ The effect estimates are adjusted for age at Women's Health Initiative Study of Cognitive Aging baseline, race/ethnicity, region of residence, employment status, household income, smoking, alcohol use, physical activity, cardiovascular disease, hypertension, hypercholesterolemia, diabetes, hormone use, history of depression prior to WHIMS baseline, and hormone therapy assignment.

### 3.6 Sensitivity analyses

A sensitivity analysis was conducted repeating the JLCMM while modeling chronological age as time instead of the years since the WHIMS‐ECHO baseline to test the robustness of our findings when time was scaled on an alternative metric. The sensitivity analyses with this alternative time metric were consistent with the main analyses suggesting that a five‐class solution best fit the data (Table S4). The estimated trajectories of depressive symptoms for each latent class (Figure S1) and associated risk of dementia from this sensitivity analysis were like the main analyses (Figure S1 Panel B and Table S5). Comparison of the BIC from the five‐class solution presented in the main analyses (BIC = 34,009) to the sensitivity analysis with the chronological age time metric (BIC = 38,466) revealed that the five‐class solution presented in the main analyses provided a better fit to the data (as indicated by the substantially lower BIC), supporting the use of the years since the WHIMS‐ECHO baseline as the time metric for the mixed‐model regression.

A second sensitivity analysis was conducted repeating the JLCMM and multinomial logistic regressions while omitting the 75 women who self‐reported a history of depression before the WHIMS baseline. The JLCMM models still identified five significant latent classes with similar trajectories and risk of dementia as described in the main results (see Figure S2 for plots of the trajectories and Table S6 for associations with dementia). After those women who self‐reported a history of depression were omitted, the women identified as having mild and stable symptoms were no longer significantly more likely to develop dementia compared to women with minimal and stable symptoms (HR = 1.64; 95% CI = 0.91, 2.07; *p* = 0.098). The decrease in the magnitude of the effect is suggestive that depression before late life may have partially driven the significant association between mild and stable depressive symptoms and dementia risk reported in the full analysis. Results of the multinomial logistic regression omitting the 43 women in the sMRI subsample who had a self‐reported history of depression before the WHIMS baseline are presented in Table S7. Following omission of those with a history of depression, the effect of WM‐SVID (OR = 1.28; 95% CI = [0.95, 1.72]; *p* = 0.109) and AD‐PS (OR = 1.36; 95% CI = [0.97, 1.91]; *p* = 0.076) on the increased risk of reporting mild and stable symptoms relative to minimal and stable were attenuated and no longer statistically significant.

## 4 DISCUSSION

In this longitudinal study conducted on a geographically diverse cohort of older women who completed as many as 19 assessments of depressive symptoms over a 21‐year study period, we identified five profiles of similar depressive symptom progression and dementia risk. Although most women reported minimal depressive symptoms across the entire study period, approximately 9% of women reported the emergence of depressive symptoms beginning in mid‐older adulthood, while 25% of women endorsed the emergence of depressive symptoms in later‐old adulthood. Women with emerging depressive symptoms, particularly during mid‐older adulthood, had the highest incidence of dementia. We also found that greater cerebrovascular disease burden was associated with depressive symptoms that began to emerge in mid‐older adulthood, while AD‐related neurodegeneration was associated with the emergence of depressive symptoms beginning in later‐older adulthood. Finally, we found that women who reported mild depressive symptoms at the study baseline that remained stable across the follow‐up were more likely to endorse a history of depression before the study baseline. These women with mild and stable symptoms were also at increased risk of dementia, greater cerebrovascular disease, and more AD‐related neurodegeneration compared to women with minimal symptoms. Sensitivity analyses revealed that the increased dementia risk, cerebrovascular disease, and AD‐related neurodegeneration associated with mild and stable symptoms were largely driven by women who reported a history of depression before older adulthood. This is the first study that we are aware of to utilize a joint latent class modeling approach to identify heterogeneous profiles of depressive symptoms and dementia risk while also examining the neuropathological underpinnings of each profile.

Our observations of increased dementia risk in individuals with emerging depressive symptoms over time are largely consistent with two prior longitudinal studies examining heterogeneous trajectories of depressive symptoms and associated dementia risk using latent growth mixture modeling approaches.^15^, ^16^ However, these studies relied on a two‐step approach where trajectories of depressive symptoms were identified in one model followed by a second analysis where the relative risk of dementia was examined over a second follow‐up period (ie, not concurrently). By using a joint modeling approach, we identified a latent class of 211 women who had delayed onset of increasing depressive symptoms in late‐older adulthood. If we had used the two‐stage approach used in prior work, we would not have identified this important group of women who are at increased risk of dementia. Another limitation of previous latent trajectory studies is the relatively short period when depressive symptoms were assessed (5 years),^15^ which may not fully capture the long preclinical phase of dementia. Although one study examined depressive symptoms over a longer time period (11 years),^16^ it only included three longitudinal assessments of depressive symptoms and was likely insensitive to the intraindividual fluctuations that are common to depressive symptoms in later life. The present study addresses both limitations by describing depression trajectories across mid‐to‐late‐older adulthood with an annual assessment of depressive symptoms over 21 years. Our findings are also consistent with another previous study using a backward time scale examining depressive symptoms over 28 years reporting increases in depressive symptoms beginning approximately 10 years before dementia onset. The current study expands on this prior work by examining heterogeneous subpopulations of women with similar progression of depressive symptom severity that differed in the magnitude of cerebrovascular disease and AD‐related neurodegeneration. Finally, previous longitudinal studies did not examine whether MRI indicators of brain health during the preclinical phase were associated with profiles of longitudinal depressive symptoms and dementia risk.

Women who reported increasing depressive symptoms during the preclinical phase of dementia in either mid‐older adulthood or late‐older adulthood had the highest incidence of dementia. This association remained robust after excluding women who had a self‐reported history of depression before older adulthood. This finding supports the hypothesis that depressive symptoms with initial onset during older adulthood may represent a prodromal signature of underlying neurologic disease. Our findings further suggest possible heterogeneity in the neurologic disease underlying depression and dementia throughout older adulthood. Women with depressive symptoms beginning to emerge in mid‐older adulthood had a greater cerebrovascular disease without increased AD‐like neurodegeneration. Women with increasing depressive symptoms beginning in late‐older adulthood had a greater burden of AD‐like neurodegeneration during mid‐older adulthood without a significantly higher cerebrovascular burden. Specifically, depressive symptoms that first emerge in mid‐older adulthood may reflect a manifestation of underlying cerebrovascular disease, whereas symptoms that emerge for the first time in late‐older adulthood may reflect underlying AD‐related neurodegeneration. The finding of increased cerebrovascular disease associated with emerging depressive symptoms in later life is consistent with a large body of literature reporting a link between vascular disease and depression in older adulthood.^6^, ^8^, ^11^

Women with mild but stable depressive symptoms throughout mid‐to‐late‐older adulthood were approximately 1.8 times more likely to develop dementia compared to women with minimal symptoms throughout the study period. Women classified in this trajectory of depressive symptoms had both significantly higher cerebrovascular burden and AD‐like neurodegeneration compared to women who had persistently minimal depressive symptoms throughout the study period. Most of the women classified in this mild and stable latent trajectory, 60%, reported a history of depression before older adulthood. Sensitivity analysis revealed that the increased risk of dementia associated with this group was primarily driven by women who had a self‐reported history of depression before older adulthood. This finding is consistent with the literature suggesting that depression, specifically with early‐life onset, might be at a two‐fold increased risk for developing dementia in older adulthood.^34^ Studies show that having a major depressive episode before older adulthood increases the risk of both AD and vascular dementia in later life.^6^ Furthermore, here we found that women reporting mild and stable symptoms had increased cerebrovascular disease burden and AD‐like neurodegeneration. This association, however, was also driven primarily by women who reported a history of depression before late life as after excluding these women associations were attenuated and not statistically significant. These sensitivity analyses support the idea that depression may exert a cumulative neurotoxic effect on the brain.^13^ Cumulative high stress may contribute to high levels of glucocorticoids, increased inflammation, and higher allostatic load, which eventually contribute to cerebrovascular disease and neurodegeneration in brain areas impacted by AD such as the hippocampus.^8^, ^13^

This study has several important clinical implications. Although there are currently no disease‐modifying treatments for dementia, the Lancet Commission's most recent report on the prevention of dementia suggests that 4% of someone's risk of dementia is explained by depressive symptoms, which are potentially modifiable.^35^ Our findings highlight the importance of longitudinal assessment of depressive symptoms over time in clinical settings to identify women who may be at increased risk of dementia during the preclinical phase. Importantly, the level of symptoms across all the latent classes that were identified was particularly low, mostly under the recommended clinical cutoff on the GDS‐15. This highlights the possibility that even a modest increase in depressive symptoms later in life may be important and associated with dementia risk. Furthermore, findings support the importance of a detailed history of past depressive episodes before older adulthood as well as the importance of stratifying late‐life depression based on the initial onset of symptoms (eg, onset before older adulthood vs onset during older adulthood). Furthermore, the initial onset of symptoms may be utilized for personalized medicine for secondary prevention efforts. For example, due to the potential neurotoxic effects of recurrent depression associated with late‐life depressive symptoms with early‐life onset, secondary prevention efforts may focus on psychotherapy interventions designed to teach strategies to manage stress, promote healthy lifestyles, and increase cognitive reserve. The goal of these efforts could be to decrease the likelihood of recurrent depressive episodes and associated neurotoxic effects on the brain. Alternatively, women with depressive symptoms that emerge for the first time in older adulthood, without evidence of depression before older adulthood, may benefit from alternative interventions. These interventions may include increased education about depression to the patient and family, medical interventions targeting the reduction of AD‐related biomarkers and cerebrovascular disease, and promotion of lifestyle factors to reduce cerebrovascular disease.

We recognize several limitations of our study. First, the women we selected for these analyses participated in both the WHISCA and WHIMS‐ECHO studies and were dementia‐free at the WHIMS‐ECHO baseline to ensure that all women were dementia‐free at the beginning of later older adulthood with an extended preclinical period where depressive symptoms were assessed. These selection criteria may limit the generalizability of our study findings. We also did not consider mild cognitive impairment (MCI) that may have accompanied depressive symptoms. Previous studies in WHIMS suggested that baseline depressive symptoms were associated with increased risk of developing MCI,^36^ and the role of MCI is unknown in our current study. Third, we cannot make inferences about how trajectories differed at baseline in sMRI variables and how trajectories of depressive symptoms were associated with changes in sMRI variables over time. Future researchers will need to utilize repeated MRI measures to examine whether there are associated changes in brain volumes based on depressive symptom trajectories. Fourth, we were unable to include random effects of the slope in our model, which might have resulted in the overextraction of latent classes.^37^ Fifth, antidepressant use has also been associated with subsequent cognitive impairment,^38^ including in the WHIMS.^39^ Future researchers will need to examine the association between starting antidepressant therapy during follow‐up and the impact of these drugs on jointly modeled trajectories of depressive symptoms and dementia risk. Future researchers will also need to expand upon the joint latent class modeling approach to consider time‐varying latent class membership. Finally, the findings are not generalizable to older men or more racially and ethnically diverse populations.

## 5 CONCLUSIONS

In conclusion, we used joint modeling approaches to characterize heterogeneity in the progression of depressive symptom severity over 21 years. Findings support the hypotheses that there is heterogeneity in the longitudinal progression of depressive symptoms and dementia risk across older adulthood. Depression with onset before older adulthood was associated with an increased risk of dementia in older adulthood, while depressive symptoms emerging for the first time in older adulthood may represent a prodromal symptom of dementia. Common pathophysiological factors, namely cerebrovascular disease, may underlie the emergence of depressive symptoms in mid‐older adulthood, while AD‐related neurodegeneration may underlie the emergence of depressive symptoms in late‐older adulthood.

## CONFLICT OF INTEREST STATEMENT

The authors have no conflicts of interest to disclose. Author disclosures are available in the supporting information.

## CONSENT STATEMENT

All human subjects provided informed consent and all procedures were approved by each institution's institutional review board (IRB).

## Supporting information

Supporting Information

Supporting Information
