# Supplementary material for: 20‐year depressive symptoms, dementia, and structural neuropathology in older women
Source: Alzheimers Dement. 2024 Apr 9;20(5):3472–84. doi: 10.1002/alz.13781 (PMC11095467; doi:10.1002/alz.13781)
Supplement: Supplementary file 1 — Supporting Information [file ALZ-20-3472-s002.pdf]

Online-only Supplemental Information: 20-year depressive symptoms, dementia, and structural neuropathology in older women

Table of Contents

Page 2: Supplemental Table S1: Model fit statistics of the joint latent class mixture models with competing risk of incident dementia and nondementia death

Page 3: Supplemental Table S2.: Class-specific estimated latent depressive symptoms (in z-score standardized units) at the Women's Health Initiative Memory Study of the Epidemiology of Cognitive Health Outcomes (WHIMS-ECHO) baseline and average linear change during the Women's Health Initiative Study of Cognitive Aging (WHISCA) and WHIMS-ECHO study periods (N = 957)

Page 4: Supplemental Table S3: Covariate effects on the level of depressive symptoms and risk of dementia from the five-class Joint Latent Class Mixture Model (N = 957)

Page 5: Supplemental Table S4: Model fit statistics of the sensitivity analyses estimating the joint latent class mixture models with competing risk of incident dementia and nondementia death with using chronological age as time instead of study years as time.

Page 6: Supplemental Figure S1: Graph of the estimated mean score of the 15-item Geriatric Depression Scale over time for each joint latent class (Panel A) and cumulative incidence of dementia (Panel B) respective for each latent class of depressive symptoms when chronological age was used as time in the mixed model regression.

Page 7: Supplemental Table S5: Sensitivity analysis examining the risk of dementia over the Women's Health Initiative Memory Study Epidemiology of Cognitive Health Outcomes (WHIMS-ECHO) study period by Identified Joint Latent Class Relative when chronological age is modeled as time compared to women with minimal symptoms throughout the Study Period (N = 957).

Page 8: Supplemental Table S6: Sensitivity analyses examining the risk of dementia over the Women's Health Initiative Memory Study Epidemiology of Cognitive Health Outcomes (WHIMS-ECHO) study period by identified joint latent class relative to women with minimal symptoms throughout the study period after excluding the 75 women who self-reported a history of depression before study baseline (N = 882).

Page 9: Supplemental Figure S2: Graph of the estimated mean score of the 15-item Geriatric Depression Scale over time for each joint latent class (Panel A) and cumulative incidence of dementia (Panel B) respective for each latent class of depressive symptoms when omitting the 75 women who self-reported a history of depression before the study baseline.

Page 10: Supplemental Table S7: Weighted* Multivariable Multinomial Logistic Regressions to Examine the Effect of White Matter Small Vessel Ischemic Disease (WM-SVID) and Alzheimer's Disease like Neurodegeneration (AD-PS) on Probability of Being Classified into Respective Joint Latent Class† (N=526).

Supplemental Table S1.
Model fit statistics of the joint latent class mixture models with competing risk of incident dementia and nondementia death[a]

Number of classes estimated	-2LL[b]	Parameters[c]	BIC[d]	Class membership	Mean posterior probabilities
One	-19370	36	38987	Class 1 = 100	Class 1 = 100
Two	-16927	42	34139	Class 1 = 68%<br>Class 2 = 32%	Class 1 = .83<br>Class 2 = .90
Three	-16887	48	34010	Class 1 = 58%<br>Class 2 = 24%<br>Class 3 = 18%	Class 1 = .81<br>Class 2 = .67<br>Class 3 = .76
Four	-16859	54	34085	Class 1 = 52%<br>Class 2 = 23%<br>Class 3 = 15%<br>Class 4 = 10%	Class 1 = .78<br>Class 2 = .64<br>Class 3 = .67<br>Class 4 = .76
Five	-16792	60	33991	Class 1 = 33%<br>Class 2 = 27%<br>Class 3 = 24%<br>Class 4 = 11%<br>Class 5 = 5%	Class 1 = .82<br>Class 2 = .81<br>Class 3 = .70<br>Class 4 = .80<br>Class 5 = .75
Six	-16781	66	34009	Class 1 = 33%<br>Class 2 = 26%<br>Class 3 = 18%<br>Class 4 = 11%<br>Class 5 = 7%<br>Class 6 = 5%	Class 1 = .75<br>Class 2 = .62<br>Class 3 = .67<br>Class 4 = .80<br>Class 5 = .77<br>Class 6 = .78

[a]all models adjust for age at initial WHISCA assessment, education, race/ethnicity, region of residence, and household income

[b]-2LL = negative log likelihood from respective model

[c]Parameters = number of parameters in each respective model

[d]BIC = Bayesian Information Criterion

Supplemental Table S2.
Class-specific estimated latent depressive symptoms (in z-score standardized units) at the Women's Health Initiative Memory Study of the Epidemiology of Cognitive Health Outcomes (WHIMS-ECHO) baseline and average linear change during the Women's Health Initiative Study of Cognitive Aging (WHISCA) and WHIMS-ECHO study periods (N = 957)

	WHIMS-ECHO baseline symptoms		Linear slope WHISCA		Linear slope WHIMS-ECHO	
Joint Latent Class	β	p	β	p	β	p
Minimal & stable (n=292)	0.00	Ref	.003	.731	**.018**	**.044**
Mild & stable (n=238)	**2.01**	**<.001**	**.039**	**.001**	**.041**	**.014**
Relapsing and remitting (n=42)	.28	.356	**-.244**	**<.001**	**.145**	**<.001**
Emerging mid-older adulthood (n=99)	**1.65**	**<.001**	**.215**	**<.001**	**.236**	**<.001**
Emerging late-older adulthood (n=211)	.20	.326	.019	.259	**.229**	**<.001**

[a] The effect estimates are adjusted for age at WHISCA baseline, race/ethnicity, region of residence, household income, and education.

Bolded estimated denote p<.05

Supplemental Table S3.
Covariate effects on the level of depressive symptoms and risk of dementia from the five-class Joint Latent Class Mixture Model (N = 957)

Covariate*	Covariate effect on level of depressive symptoms Est[†]	p
WHISCA Age on intercept	.008	.431
Non-Hispanic White	-.380	<.001
Region of residence		
Northeast	Ref	Ref
South	.157	.143
Midwest	-.008	.926
West	-.046	.621
Household income		
Less than $19,999	Ref	Ref
$20,000 to $34,999	-.123	.196
$35,000 to $49,999	**-.274**	**.007**
$50,000 to $74,999	**-.315**	**.004**
$75,000 or more	-.166	.174
Missing or don't' know	-.095	.639
Education		
Less than high school	Ref	Ref
High school	-.055	.771
More than high school	-.126	.486

	Covariate effect on risk of dementia HR	p
	Ref	Ref
WHISCA Age on intercept	**1.107**	**<.001**
Non-Hispanic White	.785	.317
Region of residence		
Northeast	Ref	Ref
South	1.156	.565
Midwest	1.228	.303
West	1.081	.722
Household income		
Less than $19,999	Ref	Ref
$20,000 to $34,999	**1.747**	**.019**
$35,000 to $49,999	1.468	.127
$50,000 to $74,999	1.401	.228
$75,000 or more	1.340	.279
Missing or don't' know	1.374	.524
Education		
Less than high school	Ref	Ref
High school	.788	.546
More than high school	.661	.269

CI = Confidence Interval

Bolded estimated denote p<.05

Supplemental Table S4.
Model fit statistics of the sensitivity analyses estimating the joint latent class mixture models with competing risk of incident dementia and nondementia death with using chronological age as time instead of study years as time

Number of classes estimated	-2LL[b]	Parameters[c]	BIC[d]	Class membership	Mean posterior probabilities
One	-19390	35	39021	Class 1 = 100	Class 1 = 100
Two	-19167	41	38616	Class 1 = 71%<br>Class 2 = 29%	Class 1 = .81<br>Class 2 = .90
Three	-19121	47	38565	Class 1 = 71%<br>Class 2 = 25%<br>Class 3 = 4%	Class 1 = .79<br>Class 2 = .78<br>Class 3 = .88
Four	-19073	53	38510	Class 1 = 65%<br>Class 2 = 15%<br>Class 3 = 13%<br>Class 4 = 7%	Class 1 = .79<br>Class 2 = .73<br>Class 3 = .74<br>Class 4 = .74
Five	-19034	59	38466	Class 1 = 31%<br>Class 2 = 28%<br>Class 3 = 27%<br>Class 4 = 10%<br>Class 5 = 3%	Class 1 = .79<br>Class 2 = .78<br>Class 3 = .78<br>Class 4 = .70<br>Class 5 = .82

[a]all models adjust for education, race/ethnicity, region of residence, and household income

[b]-2LL = negative log likelihood from respective model

[c]Parameters = number of parameters in each respective model

[d]BIC = Bayesian Information Criterion

Supplement Figure S1. Graph of the estimated mean score of the 15-item Geriatric Depression Scale over time for each joint latent class (Panel A) and cumulative incidence of dementia (Panel B) respective for each latent class of depressive symptoms when chronological age was used as time in the mixed model regression.

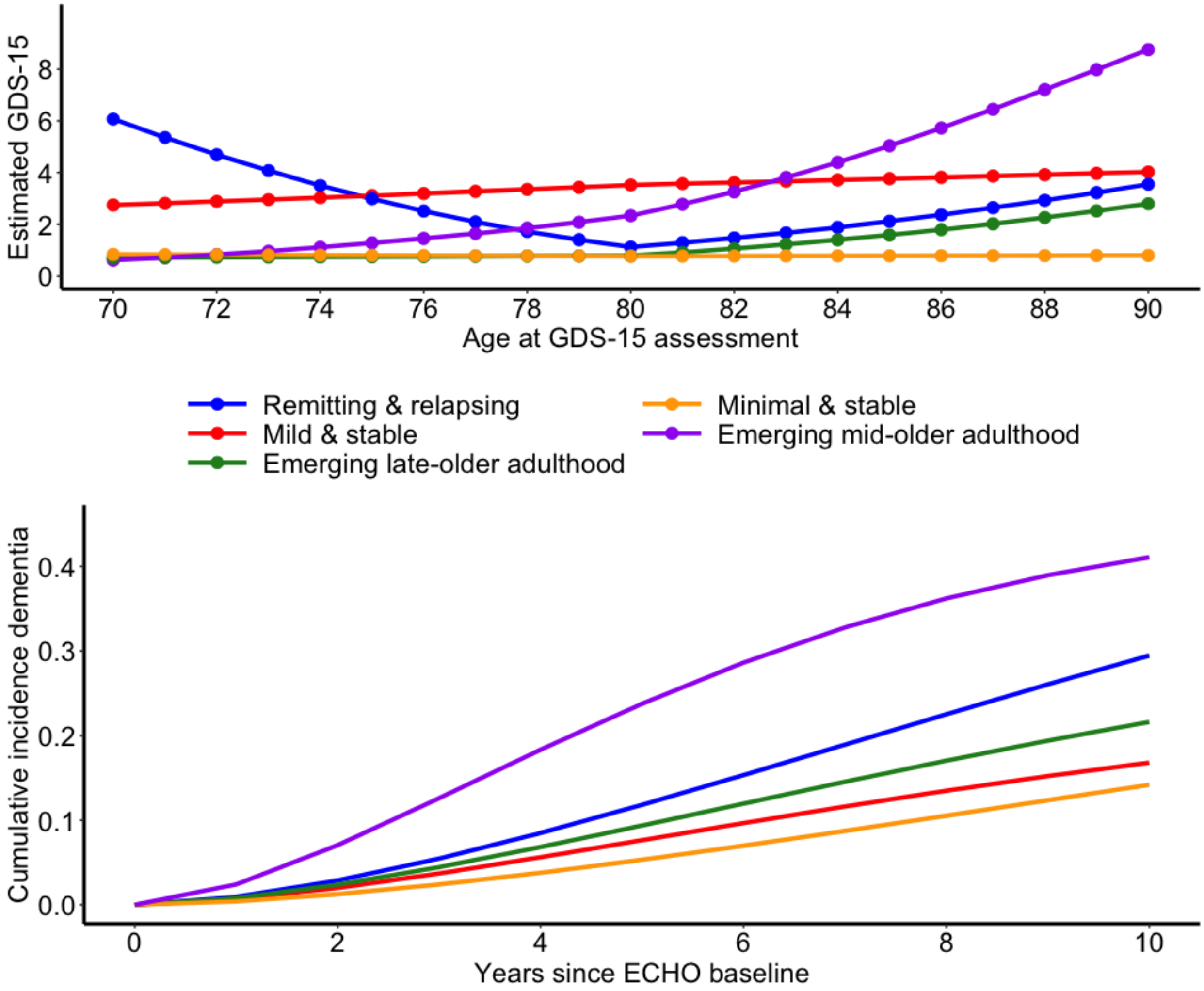

Supplemental Table S5.
Sensitivity analysis examining the risk of dementia over the Women's Health Initiative Memory Study Epidemiology of Cognitive Health Outcomes (WHIMS-ECHO) study period by Identified Joint Latent Class Relative when chronological age is modeled as time compared to women with minimal symptoms throughout the Study Period (N = 957)

		Outcome: Incident Dementia		
Joint Latent Class*	% (n)	HR†	95% CI	p
Minimal & stable (n=288)	16% (47)	Ref	Ref	Ref
Mild & stable (n=264)	19% (50)	1.65	[.99, 3.37]	.092
Relapsing and remitting (n=29)	28% (8)	2.30	[.88, 5.03]	.070
Emerging mid-older adulthood (n=85)	**32% (27)**	**6.09**	**[2.63, 9.88]**	**<.001**
Emerging late-older adulthood (n=291)	**21% (61)**	**1.94**	**[1.48, 5.19]**	**.029**

*Group membership derived from the joint latent class model examining trajectories of depressive symptoms and competing risks of dementia and nondementia mortality.

† The effect estimates in the partially adjusted model are adjusted for age at WHISCA baseline, race/ethnicity, region of residence, household income, and education.

CI = Confidence Interval
Bolded estimated denote p<.05

Supplemental Table S6.
Sensitivity analyses examining the risk of dementia over the Women's Health Initiative Memory Study Epidemiology of Cognitive Health Outcomes (WHIMS-ECHO) study period by identified joint latent class relative to women with minimal symptoms throughout the study period after excluding the 75 women who self-reported a history of depression before study baseline (N = 882)

		Outcome: Incident Dementia		
Joint Latent Class*	% (n)	HR†	95% CI	p
Minimal & stable (n=292)	11% (33)	Ref	Ref	Ref
Mild & stable (n=238)	18% (42)	1.64	[.91, 1.07]	.098
Relapsing and remitting (n=42)	26% (11)	2.16	[.86, 1.70]	.102
Emerging mid-older adulthood (n=99)	**29% (29)**	**4.02**	**[2.04, 7.90]**	**<.001**
Emerging late-older adulthood (n=211)	**29% (61)**	**2.58**	**[1.35, 4.93]**	**.004**

*Group membership derived from the joint latent class model examining trajectories of depressive symptoms and competing risks of dementia and nondementia mortality.

† The effect estimates in the partially adjusted model are adjusted for age at WHISCA baseline, race/ethnicity, region of residence, household income, and education.

CI = Confidence Interval
Bolded estimated denote p<.05

Supplemental Figure S2. Graph of the estimated mean score of the 15-item Geriatric Depression Scale over time for each joint latent class (Panel A) and cumulative incidence of dementia (Panel B) respective for each latent class of depressive symptoms when omitting the 75 women who self-reported a history of depression before the study baseline.

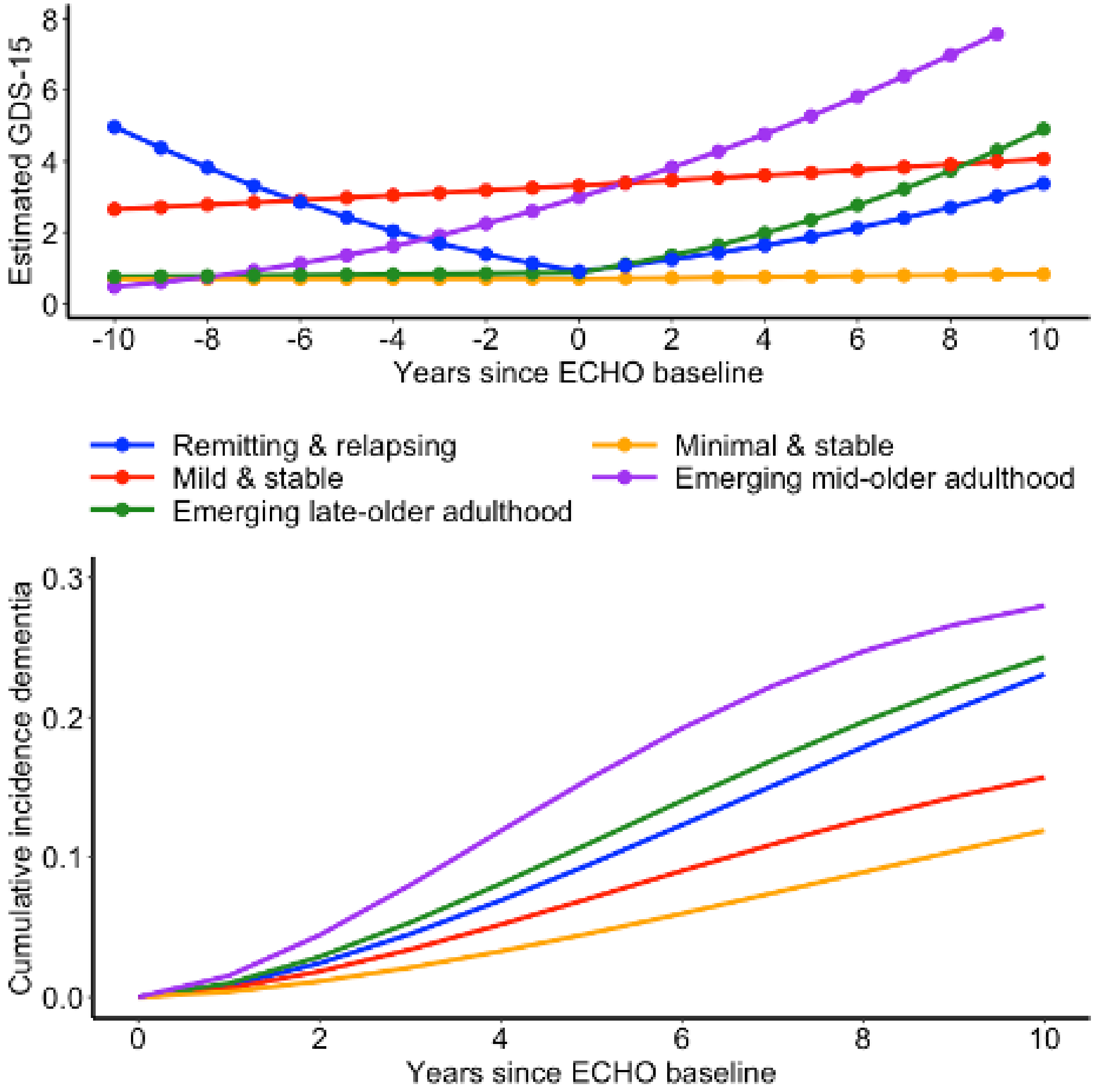

Supplemental Table S7.
Weighted* Multivariable Multinomial Logistic Regressions to Examine the Effect of White Matter Small Vessel Ischemic Disease (WM-SVID) and Alzheimer's Disease like Neurodegeneration (AD-PS) on Probability of Being Classified into Respective Joint Latent Class† (N=526).

Outcome: Joint Latent class membership	WM-SVID Mean (SD)	WM-SVID effect per one SD			AD-PS Mean (SD)	AD-PS effect per one SD		
		OR‡	95% CI	p		OR‡	95% CI	P
Minimal & stable (n=196)	2.63 (3.68)	1.00	Ref	Ref	.23 (.16)	1.00	Ref	Ref
Mild & stable (n=136)	3.91 (5.03)	1.28	[.95, 1.72]	.109	.29 (.19)	1.36	[.97,1.91]	.076
Relapsing and remitting (n=28)	2.19 (3.68)	.61	[.24, 1.52]	.290	.30 (.20)	1.68	[.94, 3.02]	.080
Emerging mid-older adulthood (n=52)	4.52 (6.15)	1.47	**[1.03, 2.11]**	**.036**	.29 (.15)	1.29	[.80, 2.07]	.292
Emerging late-older adulthood (n=114)	3.01 (3.35)	1.06	[.75, 1.49]	.737	.34 (.22)	**1.83**	**[1.30, 2.59]**	**<.001**

* To account for the uncertainty in latent class membership, the posterior probability of latent class membership was included as a weight in the multivariable multinomial logistic regression.

† Group membership derived from the joint latent class model examining trajectories of depressive symptoms and competing risks of dementia and nondementia mortality.

‡ The effect estimates are adjusted for age at WHISCA baseline, education, race/ethnicity, region of residence, employment status, household income, smoking, alcohol use, physical activity, cardiovascular disease, hypertension, hypercholesterolemia, diabetes, hormone use, and hormone therapy assignment.

OR = Odds ratio
CI = Confidence Interval
WM-SVID = White matter small-vessel ischemic disease
AD-PS = Alzheimer's disease pattern similarity score
Bolded terms denote p < .05
